# Acute cell viability and nitric oxide release in lateral menisci following closed-joint knee injury in a lapine model of post-traumatic osteoarthritis

**DOI:** 10.1186/1471-2474-15-297

**Published:** 2014-09-06

**Authors:** Megan L Killian, Roger C Haut, Tammy L Haut Donahue

**Affiliations:** Department of Mechanical Engineering, Colorado State University, 1374 Campus Delivery, Fort Collins, CO USA; Orthopaedic Biomechanics Laboratories, Michigan State University, East Lansing, MI USA; Department of Orthopaedic Surgery, Washington University School of Medicine, St. Louis, MO USA; Department of Mechanical Engineering, Michigan Technological University, Houghton, MI USA

**Keywords:** Meniscus, Trauma, Knee, Cell viability, Anterior cruciate ligament

## Abstract

**Background:**

Traumatic impaction is known to cause acute cell death and macroscopic damage to cartilage and menisci *in vitro.* The purpose of this study was to investigate cell viability and macroscopic damage of the medial and lateral menisci using an *in situ* model of traumatic loading. Furthermore, the release of nitric oxide from meniscus, synovium, cartilage, and subchondral bone was also documented.

**Methods:**

The left limbs of five rabbits were subjected to tibiofemoral impaction resulting in anterior cruciate ligament (ACL) rupture and meniscal damage. Meniscal tear morphology was assessed immediately after trauma and cell viability of the lateral and medial menisci was assessed 24 hrs post-injury. Nitric oxide (NO) released from joint tissues to the media was assayed at 12 and 24 hrs post injury.

**Results:**

ACL and meniscal tearing resulted from the traumatic closed joint impact. A significant decrease in cell viability was observed in the lateral menisci following traumatic impaction compared to the medial menisci and control limbs. While NO release was greater in the impacted joints, this difference was not statistically significant.

**Conclusion:**

This is the first study to investigate acute meniscal viability following an *in situ* traumatic loading event that results in rupture of the ACL. The change in cell viability of the lateral menisci may play a role in the advancement of joint degeneration following traumatic knee joint injury.

**Electronic supplementary material:**

The online version of this article (doi:10.1186/1471-2474-15-297) contains supplementary material, which is available to authorized users.

## Background

Anterior cruciate ligament (ACL) injury is one of the most common injuries to the knee[1]. Concomitant injuries, such as meniscal tears and bone bruising, often occur with traumatic ACL rupture[1]. These injuries are known to play a role in soft tissue damage and the progression of post-traumatic osteoarthritis (PTOA) of the knee. Acute morphological and biological perturbations, such as cartilage fissuring, subchondral bone bruising, and chondrocyte death have been associated with rapid joint degeneration[2]. Such injuries, along with posterolateral meniscal tearing, have been observed after traumatic impaction that leads to ACL rupture[3, 4].

Traumatic impaction has been shown to induce acute cell death and macroscopic damage to cartilage and menisci *in vitro*[5, 6]. Chondrocyte cell death following cartilage injury has been associated with increased expression of pro-inflammatory cytokines and increased release of nitric oxide (NO) in such studies. High levels of NO can lead to increased apoptosis in chondrocytes[7] and synoviocytes[8], and increased NO levels have been associated with the progression of osteoarthritis. Additionally, *in vitro* cyclic mechanical loading has been shown to play a role in NO release from joint tissues such as the meniscus[9] and cartilage[10]. Previous studies have looked at NO release in a lapine model following transection of the anterior cruciate ligament[11] and following partial meniscectomy[12]. These studies demonstrated an increase in NO production from meniscal tissue following prolonged ligament and meniscal injury, however these studies did not replicate PTOA via traumatic compressive loading, nor were the acute changes to the menisci documented. Understanding how the menisci behave in the acute phase following traumatic joint impaction may help guide future therapies following traumatic knee injury in order to prevent the development of PTOA.

The purpose of this study was to investigate tear morphology, cell viability in the medial and lateral menisci and investigate the release of NO from joint tissues following traumatic impaction of an *in situ*, closed-joint animal model. Specifically, it was hypothesized that traumatic impaction would result in decreased cell viability compared to control joint tissues. We expected increased cell death in the lateral menisci (LM) compared to medial menisci (MM) following traumatic impaction based on the higher risk of LM tearing following this type of joint impaction[4]. Additionally, it was hypothesized that the impacted joints would demonstrate a greater release of NO than control tissues.

## Methods

### Traumatic impaction

**Figure 1:**
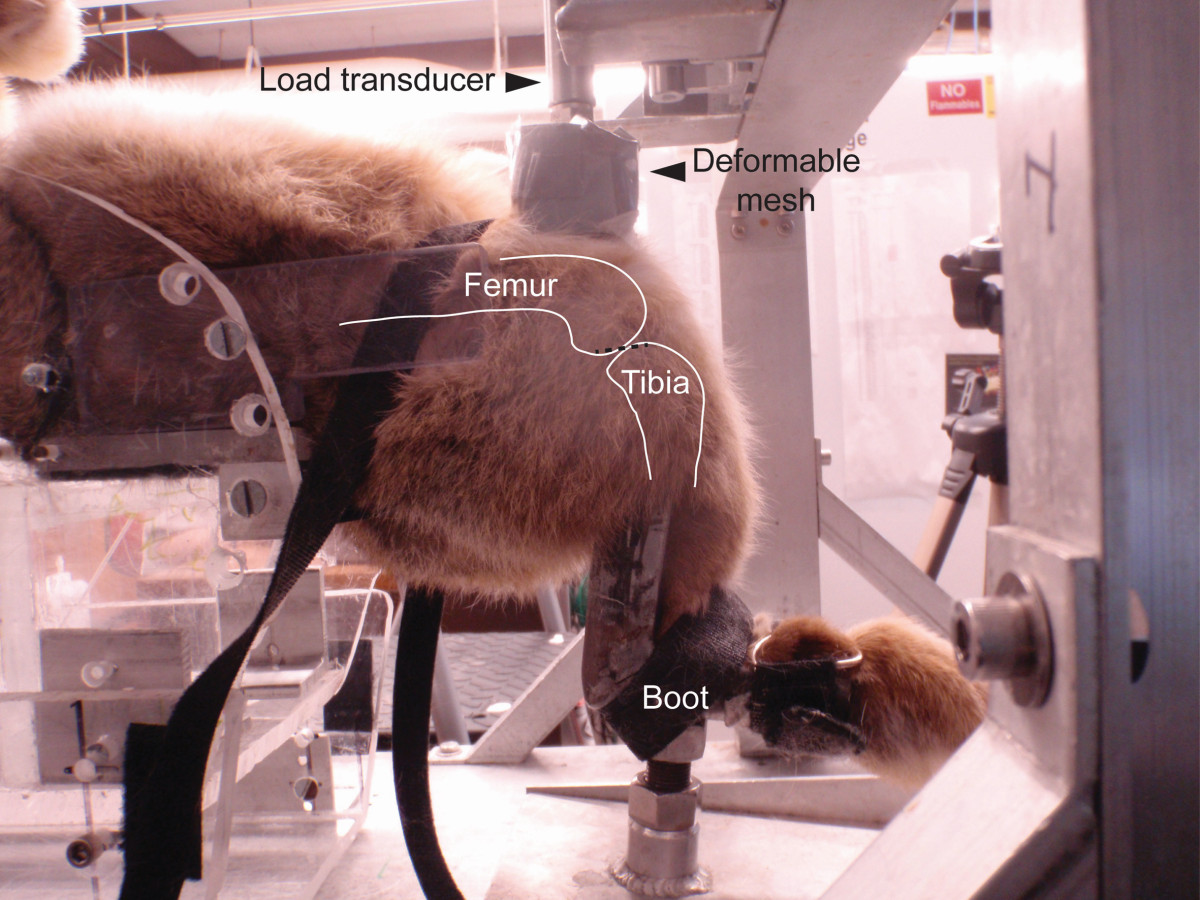
**Impact experiments were performed by dropping a gravity-accelerated mass onto the flexed tibial-femoral joint.** The rabbit knee was positioned such that the deformable head struck the distal femur in order to induce anterior tibial subluxation, ACL rupture and meniscal tearing.

Five skeletally mature Flemish Giant rabbits (5.9 ± 0.8 kg) were used in the study following approval by the Institutional Animal Care and Use Committee. The animals were housed in individual cages (61x122x46 cm^3^) prior to the study. Animals were tranquilized with 1 mg/kg acepromazine and anesthetized using 5% isoflorane and 1% oxygen. The animals were euthanized by overdose of isoflorane and intracardial injection of potassium chloride. Immediately after death, the animals received a blunt force insult to the left tibiofemoral (TF) joint using a previously described drop tower[4]. The drop tower sled was arrested electronically after one impact. A pre-crushed, deformable aluminum impact interface (Hexcel, Stamford, CT, 3.76 MPa crush strength) was used to ensure uniform loading over the femur. The interface was mounted in front of a 4.45 kN (1000 lb) load transducer (ICP® force sensor, model 208C04, PCB Electronics, Depew, New York, USA) (Figure 1). The impact mass was 1.75 kg and it was dropped from 0.88 m. The animal was laid supine with the knee flexed at 90 deg. The foot was fixed in a boot with two Velcro straps. Two additional Velcro straps crossed over the femur. The left leg was positioned such that the dropped mass struck the distal femur, which resulted in a “kissing” impaction of the femur onto the posterior tibial plateau (Figure 1). The impact was such that load is transferred through the patella to the femur, and through the femur to the tibia. The patella was in line with the impact area and its imprint was always present in the deformable interface. No damage was noted to the patella in any of the impacted limbs. This impaction resulted in anterior tibial subluxation. After impaction, an anterior drawer test was used to diagnose potential ACL tears. Briefly, an examiner (MLK) held the tibia and pulled anteriorly and visually assessed translation of the tibia with respect to the femur. The right leg served as a non-impacted, internal control.

Immediately following impaction (within ~1 minute), both impacted and control limbs were disarticulated at the knee and dissected under sterile conditions. Knees were disarticulated and the presence and location of meniscal tears, as well as integrity of the ACL, was determined. The tibial plateau and femoral condyles were then harvested approximately 0.6-0.8 cm from the proximal and distal ends, respectively. Menisci remained attached to tibial plateaus. Once removed, tibiae (T) and femur (F) blocks were rinsed in PBS and growth media (44% Hams F12, 44% Dulbecco’s Modified Eagle Medium (DMEM), 10% fetal bovine serum, and 2% penicillin/streptomycin; 37°C). Within 1 hour post-impaction, whole tibiae and femur blocks were submerged in individual wells containing 10 mL growth media and incubated at 37°C and 5% CO_2_. Media was removed at 12 hrs post-harvest (t_12_) and replaced with fresh media. Media was again removed at 24 hrs post-harvest (t_24_).

### Tibiofemoral contact pressures

To maintain integrity of all structures, including the synovium and skin, prior to impaction, contact pressure data was collected for only one animal because it required an incision and opening of the joint prior to impaction. One animal (A5) was used to document contact pressures in the knee during impaction using a previously described protocol[13]. Packets of stacked, medium and low range, pressure sensitive film (Prescale, Fuji Film Ltd., Tokyo, Japan) were inserted through the joint, anterior to posterior between the femoral condyles and the menisci, to record contact pressure in the joint during impaction. After impact, the films were removed from the packet and scanned (Scanmaker MRAS-1200E6, Microtek, Taiwan). The medium range film was digitized (Photostyler v1.1A, Aldus Co., Seattle, WA). Total area of contact was calculated from markings on the pressure film. A custom MATLAB (Mathworks, Natick, MA) script was used to determine color intensity of pixels, ranging from white (no pressure) to red (high pressure), and calibrated in a material test system using known pressures. Pressure was averaged across the film and peak pressures were determined as well as the contact area in each tibial compartment having pressures over 20 MPa.

### Cell viability

At t_24_, menisci were removed from tibial plateaus and 2-3 parallel 150 μm thick coronal sections from the central regions of the lateral (LM) and medial (MM) menisci were obtained. Maximal cell death is observed after 24 hr of culture, and therefore this period of incubation was used[13]. If a meniscal tear was present, the slices included the tear, while remaining as centrally located as possible. Sections of menisci were immediately incubated in 2 mM ethidium homodimer-1 and 4 mM Calcein AM (Life Technologies, Grand Island, NY) in PBS and incubated for 30-45 min in the dark at room temperature. Images were captured for both live (green fluorescence) and dead (red fluorescence) cells using a fluorescence microscope (Olympus AX70 microscope and DP70 camera, Center Valley, PA) at the same magnification and exposure (10X). CellC was used to count live and dead cells from individual field of view images[14], where the percentage of cell viability was equal to the amount of live cells divided by the total sum of dead and live cells. Data from the 2-3 150 μm-thick slices for each menisci were averaged for four of the five animals in the study.

### Nitric oxide release

The release of NO into the tibial and femoral culture media at t_12_ and t_24_ was quantified using a commercially available assaying kit (Nitrate/Nitrite Colorimetric Assay Kit, Cayman Chemical Company, Ann Arbor, MI). Conditioned media were filtered using a 10 kD cut-off filter (Millipore Microcon YM-10, Bedford, MA, USA) prior to performing nitrate/nitrite assays. The final concentration was normalized to wet weight (g) of the tissue (N = 3 for each time point).

### Statistics

Statistical analysis was performed using Prism (Graphpad 6.0d). Wilcoxon-signed ranks tests were used to determine statistical differences in cell viability between control and impacted limbs for lateral menisci (LM) and medial menisci (MM). Descriptive statistics were used to compare NO release from control and impacted joint tissue after 12 and 24 hrs of incubation. P values < 0.05 were used for all analyses.

## Results

**Figure 2:**
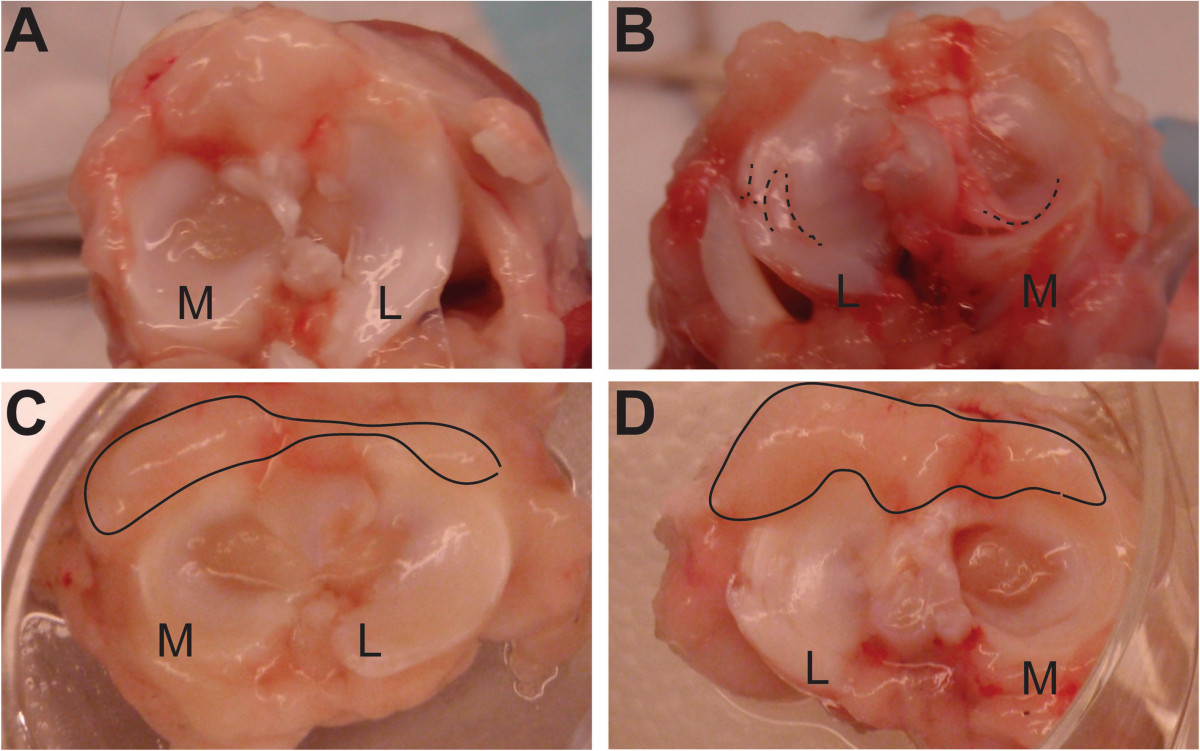
**Gross morphology of menisci showed tears and synovial swelling at 24hr post-impaction.** Gross morphology of menisci immediately after dissection in **(A)** control knee and **(B)** impacted left knee resulting in ACL tear. Dashed lines highlight tears in the posterior region of both menisci. Synovial swelling after 24 hrs incubation was generally greater for **(D)** impacted knees compared to **(C)** control limbs. L = lateral, M = medial.

Meniscal tears and ACL rupture for each animal is described in Table 1. The impact force to induce ACL rupture and meniscal tearing was 737 ± 96 N (mean ± SD). Four of the five animals experienced ACL damage, although all five were diagnosed with a characteristic “clicking” noise during anterior drawer tests prior to dissection. Control limbs did not demonstrate either meniscal or ACL tears at the time of dissection (Figure 2A). Following traumatic impaction, four of five animals experienced LM tears, primarily longitudinal or radial tears in the central-posterior region. One animal developed a LM tear without ACL rupture and two of the five animals had MM tears with concomitant ACL injury following traumatic impaction. One animal experienced both LM and MM tears with an ACL tear following impaction (Figure 2B). All meniscal tears were located in the central and/or posterior regions (Figure 2B) with two tears having full-width LM radial tears. Qualitative assessment of synovial tissue indicated that the parameniscal regions of the impacted tissue, following a 24 hr period of incubation, indicated increased tissue swelling compared to control tissue (Figure 2C&D).

**Table 1:** **Meniscal tears and ACL rupture descriptions**

Animal	Sex	Weight (kg)	Impact force (N)	Tear description
1	M	5.1	727	ACL tear, medial and lateral meniscal tears
2	M	6.0	672	NO ACL tear, but did have lateral posterior meniscal tear
3	M	5.4	890	Partial ACL tear, lateral posterior meniscal tear
4	F	7.1	661	ACL tear, medial meniscus tear in posterior region
5	F	5.8	733	ACL tear, lateral meniscal tear

**Figure 3:**
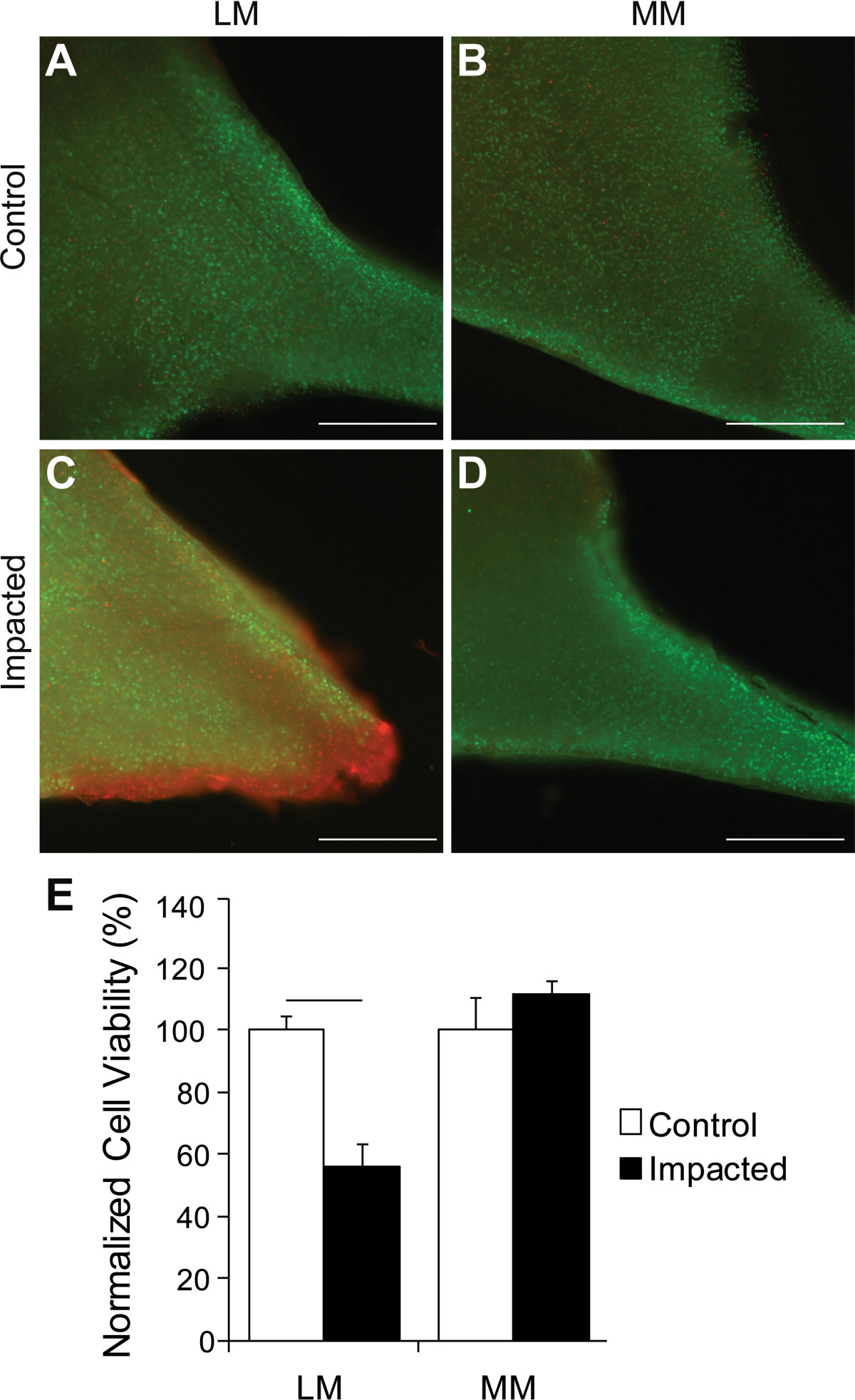
**Cell viability was reduced at meniscal tear sites. A-D)** Representative cell viability images of LM **(A & C)** and MM **(B & D)** from control (top panel) and impacted (middle panel) knees. Increased cell death (red cells) was appreciable at tear edge of the impacted LM. **E)** Cell viability of impacted limbs was significantly reduced in LM with impaction compared to control. Data is shown as percentage of cell viability of control or impacted limbs normalized to the percentage of cell viability of control limbs. Bar indicates a strong statistical trending decrease in cell viability (p = 0.06). Scale bar = 500 μm.

Measurement of contact pressures in a single knee joint showed that the average contact pressure generated in the lateral and medial compartments of the knee were 27.5 MPa and 22.7 MPa, respectively. The peak contact pressures in the lateral and medial compartments were 50.9 MPa and 48.4 MPa, respectively. The contact area with pressures over 20 MPa were 19.7 mm^2^ on the lateral plateau and 18.5 mm^2^ on the medial plateau.From a qualitative standpoint, uninjured controls demonstrated consistent and uniform cell viability across meniscal sections, with some dead cells evenly distributed across the sections (Figure 3A&B). In menisci from impacted limbs, cell death was most apparent along the torn edges (Figure 3C). Impaction led to a strong statistical trending decrease (p = 0.06) in cell viability in the LM compared to the same menisci of controls (Figure 3D). Changes in cell viability of the MM were not statistically different between control and impacted limbs (Figure 3E).

**Figure 4:**
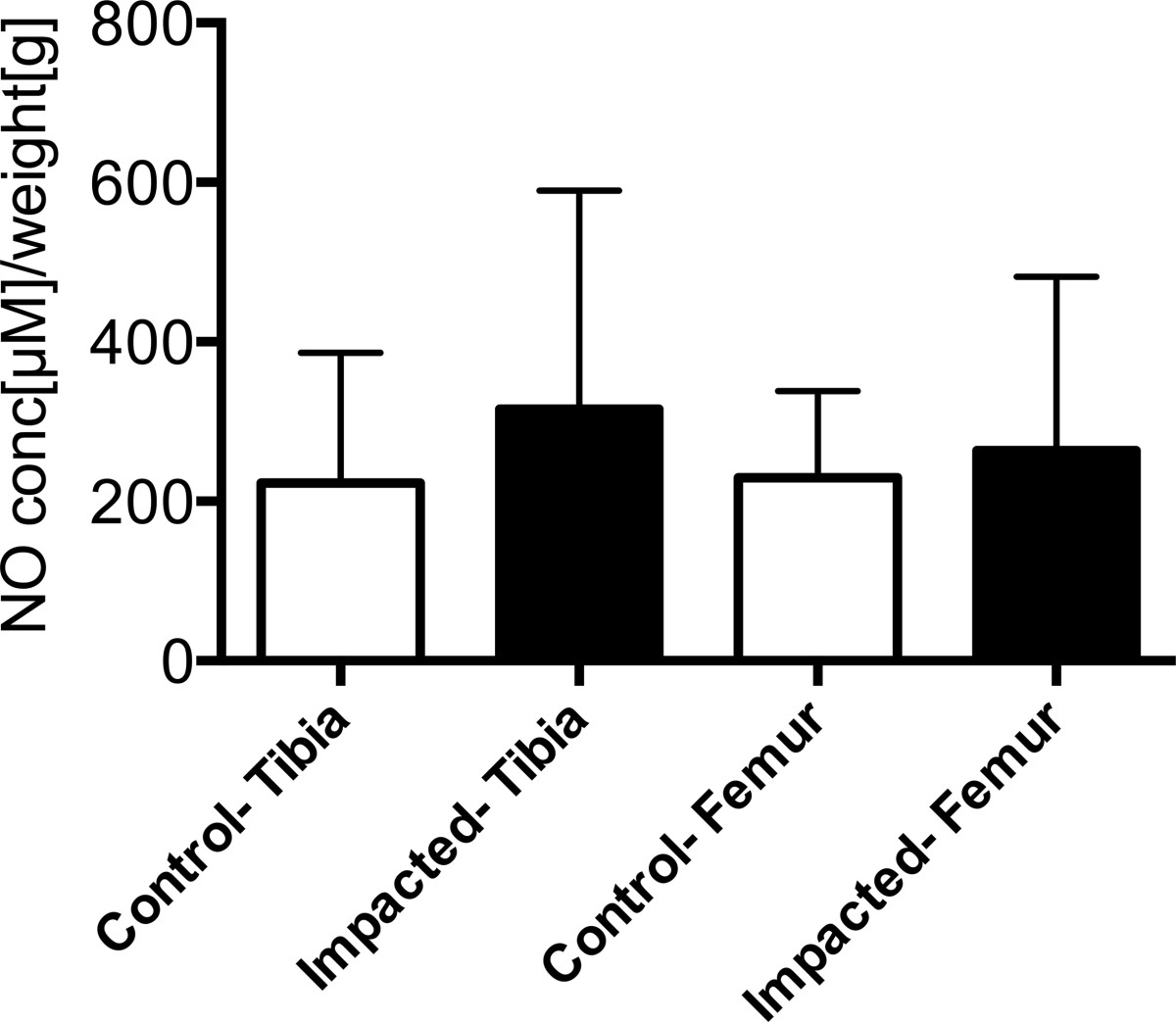
**Cumulative NO release of meniscotibial (T) and femoral (F) tissue from control and impacted limbs measured in the culture media in the first 24 hrs after impaction.** NO concentration (μM) was normalized to wet weight of tissue (g). Mean ± 95% confidence intervals.

Cumulative NO following traumatic impaction was highly variable (162 ± 104 μM/weight[g] femur, 64% confidence interval (CI); 169 ± 126 μM/weight[g]/ tibiae 74% CI) compared to control limbs (110 ± 30 μM/weight[g] femur, 27%CI; 112 ± 47 μM/weight[g] tibiae, 42% CI) after 24 hrs (Figure 4). Similar results were present in the t_12_ samples from the tibia and femur. Variability in NO release was 64-83%CI for impacted joints and 14-42%CI for control joints.

## Discussion

This is the first study to explore the role of *in situ* loading on meniscal cell viability, specifically involving traumatic impaction, ACL rupture, and acute meniscal tearing. Prior to the current study, viability of meniscal cells following traumatic loading has only been explored *in vitro* using explants and tissue culture in a limited number of studies[6, 15]. While studying impaction *in vitro* has allowed researchers to isolate the tissue and cell populations of interest from external biological factors (e. g. synoviocytes, macrophages), it is likely that tissue culture and explant loading could alter the boundary conditions and mechanical behavior of tissues compared to that experienced in the *in situ* environment[5].

In the present study, meniscal cell death was largely localized near and along the surfaces of tears. Similar findings have been observed following *in vitro* studies on meniscal and cartilage explants[5, 6]. In the current study, cell death was most apparent in the LM, and such decreases in cell viability may be explained by the increased presence of meniscal tears in that compartment in conjunction with a suggested increase in contact pressures at the time of impaction. A decrease in cell viability may suggest the need for acute pharmacological intervention following traumatic injury. For example, treatment with cell surfactants, such as poloxamer-P188 (P188), a non-ionic surfactant, has previously demonstrated success maintaining cell viability in chondrocytes following impact loading of articular cartilage[16–19]. Such treatments may improve cell membrane stiffness and their ability to induce cellular and matrix repair following a single high-impact mechanical load[19]. At present, it is unclear whether an acute decrease in cell viability at the time of injury impairs the remodeling and healing of the injured meniscus following a traumatic impaction. Additionally, it is unknown whether meniscal cells play a role in degenerative and/or healing adaptations in the chronic setting following traumatic meniscal/ACL injury. Future work is needed to investigate potential molecular cues directing cellular activity and autophagy initiated by meniscal fibrochondrocytes.

Recently, *in vivo* studies have established the role of high-stress loading on cartilage viability of the knee using the lapine animal model[13]. Isaac et. al., measured cell death of cartilage *in vivo* using an impact protocol similar to the current study, without tibial translation in order to prevent anterior tibial subluxation. This constraint prevented ACL rupture and meniscal tears[13]. In their study, elevated chondrocyte death was observed in lateral more than the medial compartment of the knee. Their findings suggested that increased chondrocyte death in the lateral compartment was due primarily to the relatively higher levels of impact-induced contact stresses generated in the lateral versus in the medial compartment of the lapine knee, as reflected in the current study. Localization of stress and regions of high cell death are then likely correlated and dependent on the mechanism of energy transfer across the joint during traumatic loading. While it is generally understood that the medial compartment observes higher contact loads than the lateral compartment during normal loading in the human knee[20, 21], localized stresses on the articulating surfaces of the knee during traumatic loading conditions have only been marginally characterized in translatable animal models[22]. In a recent cadaveric model of ACL injury patterns, Levine et al. demonstrated that valgus rotation of the tibia prior to impact loading resulted in concomitant lateral meniscal tears in conjunction with ACL rupture[23]. Their study suggested the dependence of tibial plateau injury, but not ACL rupture, on applied loading conditions[23]. Additionally, while their study demonstrated orientation-driven injuries to the lateral meniscus, post-impaction injuries to the medial menisci were not apparent. In the present study, we demonstrate that deep-knee flexion with high impact loading on the tibiofemoral joint leads to predominantly posterolateral meniscal injuries, with and without ACL rupture, in a novel lapine model of PTOA. Future studies should focus on localized loading and contact stresses of the menisci, ACL, and tibial plateau at the time of traumatic impaction using this animal model in order to gain insight into realistic and translatable models of sports-related knee injuries.

Traumatic injury to the knee following events such as sport jumping and landing (e.g. skiing or basketball) has been shown to lead to increased axial tibia loading, and such loading can lead to increased anterior tibial forces, anterior tibial translations and internal tibial rotation[24]. In the present study, joint injury was induced by anterior tibial subluxation, which primarily led to longitudinal tears in the posterior aspect of the lateral and/or medial menisci and ACL ruptures. Anterior tibial translation and subsequent rupture of the ACL following impaction may have encouraged a combined “pinched” shearing of the menisci along with compression of the femur on the posterior edge of the tibial plateau. We speculate that the presence of meniscal and ACL tears were initiated by soft tissue compression and accumulated strain energy in these tissues which would have otherwise been attenuated through intact underlying soft tissue and bone.

Nitric oxide is a known byproduct of chondrocytes in the presence of inflammatory markers, and increased levels of NO can lead to increased chondrocyte death. Therefore, we investigated the release of NO from cartilage and subchondral bone of the femur as well as cartilage, subchondral bone and menisci from the tibia. In this study, we did not observe a statistically significant increase in NO release following traumatic impaction in the acute setting. The variability in the NO data, as indicated by high confidence variables, suggests our data are inconclusive. It is possible that there could be no difference between control and impacted limbs; however, with approximately 70%CI for impacted limbs, it is not possible to draw strong conclusions from this study. Additionally, it is also possible that acute traumatic impaction does not lead to release of NO from the meniscus or articular cartilage within 24 hours of injury, or that the en-bloc samples of cartilage, meniscus, and bone incubated together in culture media had an overwhelming effect on the detection of NO released to the media. Long term adaptations following transection of the ACL with meniscal injury may further increase NO production, as shown in other studies[11, 12]. It is possible that we did not select a longitudinal time point with sufficient duration to detect changes between control and impacted limbs. Nonetheless, data from the present study representing findings in NO release should be taken with caution.

This study is not without limitations. One limitation was the variability of ACL and meniscal injury in our sample population. It is likely that anatomical differences between animals led to differences in post-trauma outcomes in ligament and meniscal injuries. Specifically, the rabbits utilized in this study were not identical in size and weight; differences in bone architecture, fatty deposition, and muscle mass may have played a role in the degree of injury incurred following impaction. These variables were not accounted for in our study design.

Another limitation was the lack of separation between the different articular tissues in post-dissection culture. It is unclear, from the experiments performed in this study, which tissues were responsible for the release of NO and to what magnitude each individual tissue contributed. Therefore, these findings are only suggestive and do not qualify menisci, cartilage, subchondral bone, or synovium as primary donor(s) of NO following traumatic joint injury. This study merely provides a first-pass as to how the joint, as a collection of tissues, contributes to the release of NO. Future studies should isolate and culture individual tissues in order to better assess the isolated contributions of these tissues in NO release. Additionally, this study is limited by the lack of external controls in assessing meniscal cell viability with and without impaction. This study utilized contralateral limbs of impacted knees to assess cell viability and NO release. While paired comparisons provide increased statistical power, it is possible that genetic variability of the lapine population selected may have biased our findings. Lastly, this study is limited by its utilization of a lapine model to induce traumatic ACL rupture, and may not be directly translatable to the human patient. Specifically, it is not clear from this work whether our findings in a lapine model can be translated to the human case. Specifically, the joint anatomy of the lapine knee is not identical to that of the human[25], and these differences may play a role in differential joint loading biomechanics between the two species.

This study illustrated that meniscal defects associated with impaction-induced ACL rupture led to fibrochondrocyte cell death primarily localized near surface lesions in the lateral menisci within the first 24 hrs following injury. An understanding of the cellular response of meniscal tissue to traumatic impaction may lead to alterations in acute treatments that could potentially help to delay progression of meniscal degradation and development of knee joint PTOA.

## Conclusion

Traumatic impaction, resulting in meniscal tears, led to acute cell death localized to surface lesions in a rabbit model of post-traumatic OA.

## Authors’ original submitted files for images

Below are the links to the authors’ original submitted files for images. Authors’ original file for figure 1 Authors’ original file for figure 2 Authors’ original file for figure 3 Authors’ original file for figure 4
