# Supplementary figures and images for: Acute cell viability and nitric oxide release in lateral menisci following closed-joint knee injury in a lapine model of post-traumatic osteoarthritis
Source: BMC Musculoskelet Disord. 2014 Sep 6;15:297. doi: 10.1186/1471-2474-15-297 (PMC4246489; doi:10.1186/1471-2474-15-297)

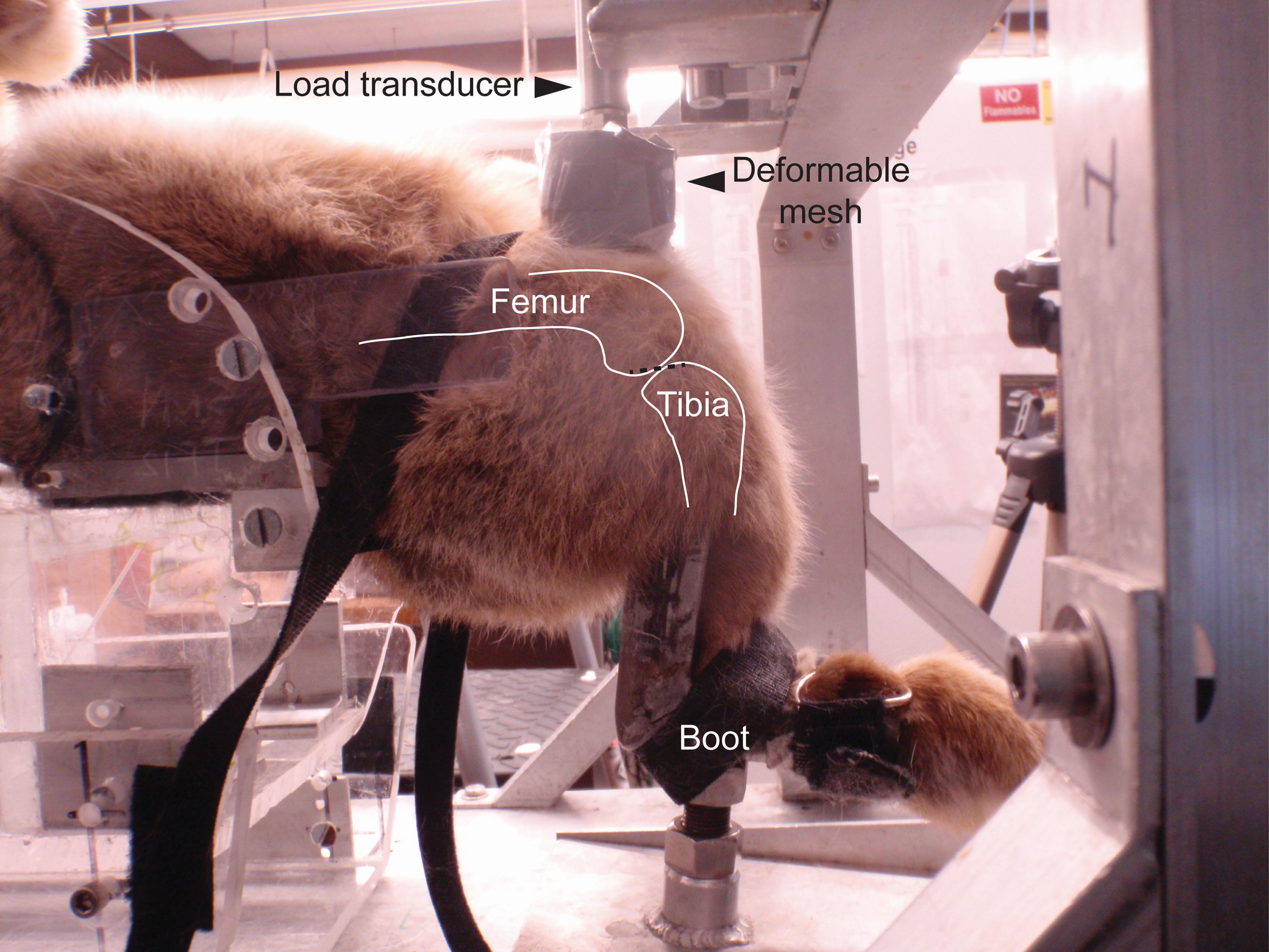

Supplement: Supplementary file 1 — Authors’ original file for figure 1 [file 12891_2013_2316_MOESM1_ESM.tif]

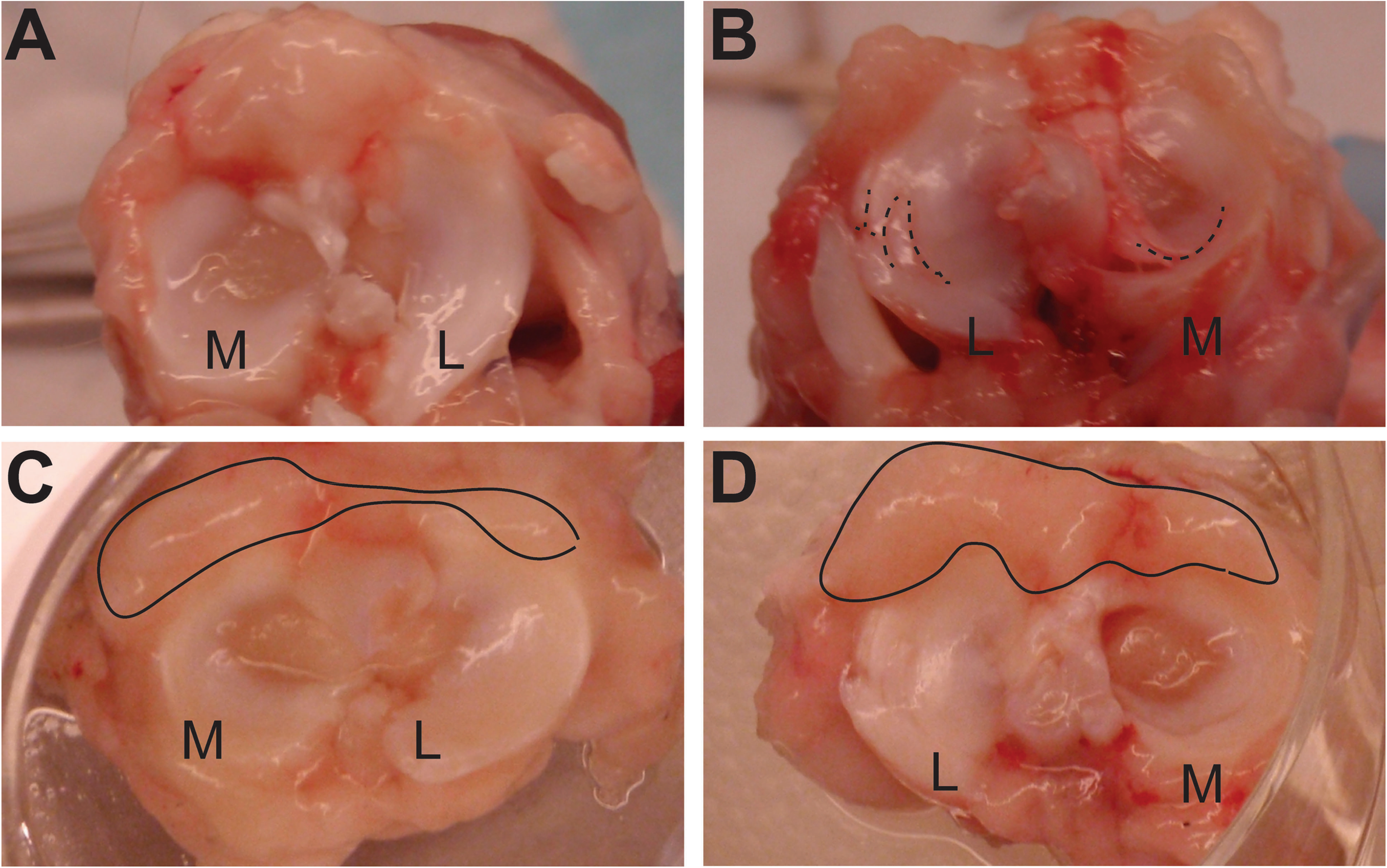

Supplement: Supplementary file 2 — Authors’ original file for figure 2 [file 12891_2013_2316_MOESM2_ESM.tif]

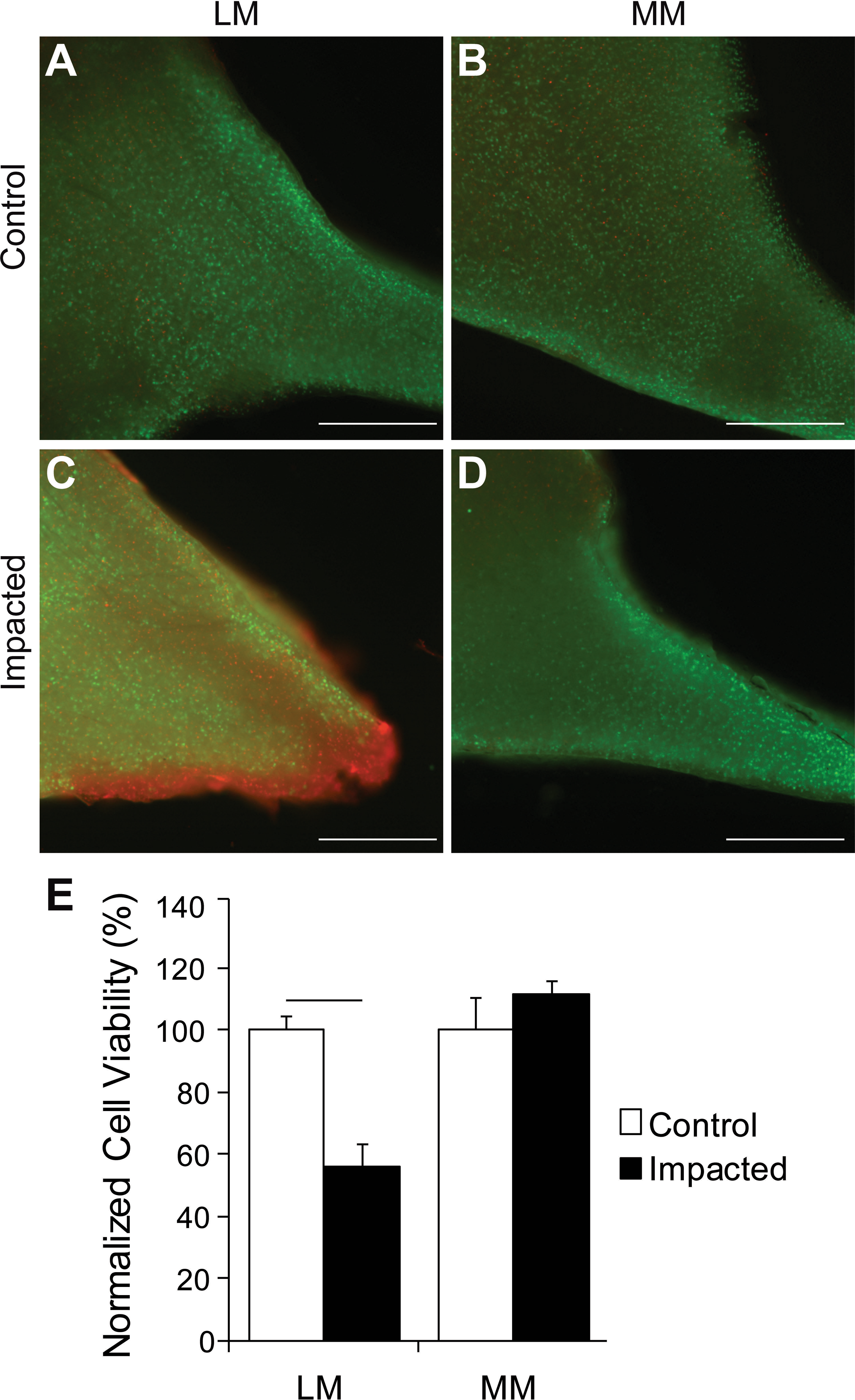

Supplement: Supplementary file 3 — Authors’ original file for figure 3 [file 12891_2013_2316_MOESM3_ESM.tif]

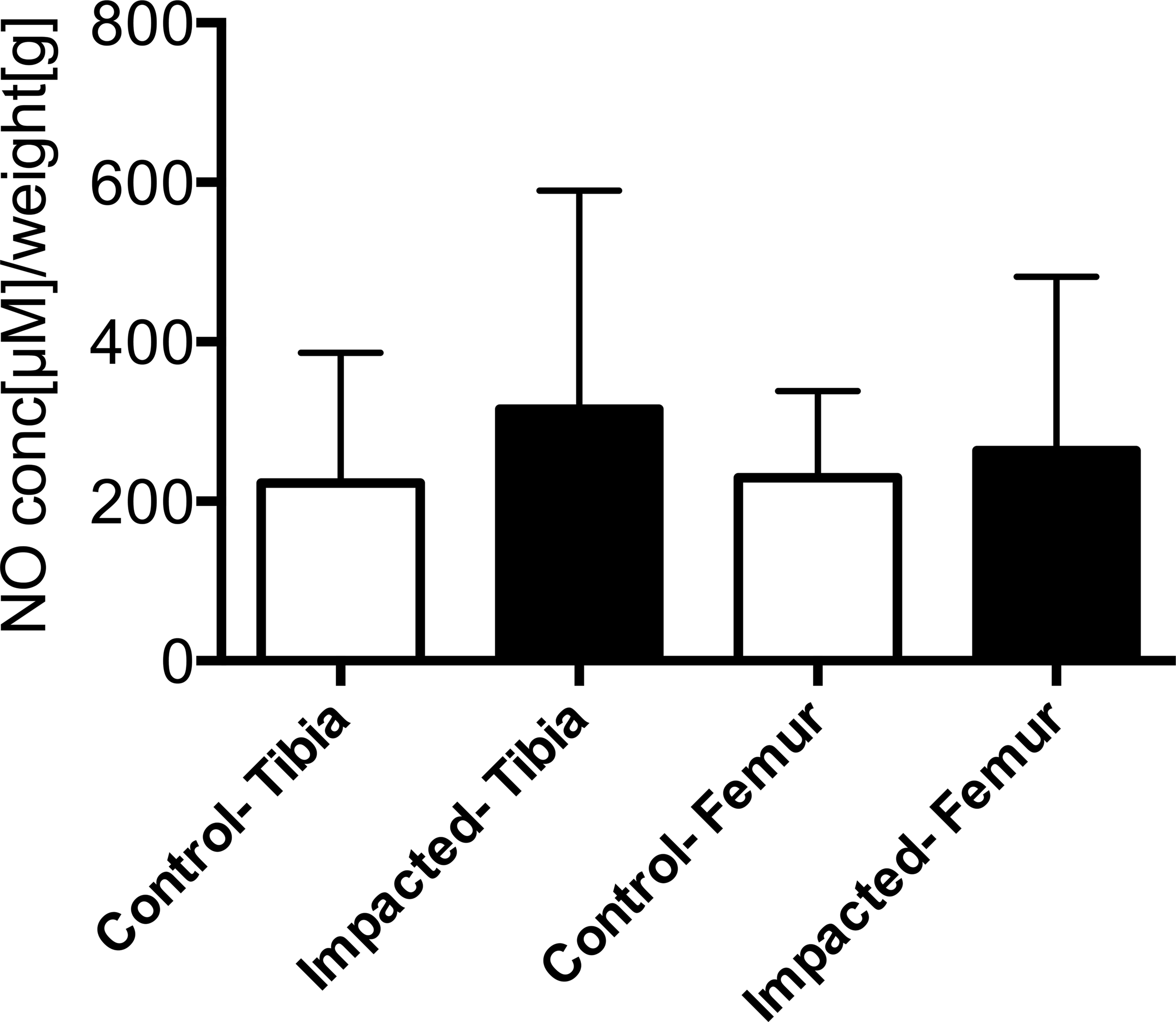

Supplement: Supplementary file 4 — Authors’ original file for figure 4 [file 12891_2013_2316_MOESM4_ESM.tif]
